# Radioactive Wastewater Treatment Technologies: A Review

**DOI:** 10.3390/molecules28041935

**Published:** 2023-02-17

**Authors:** Hailing Ma, Minghai Shen, Yao Tong, Xiao Wang

**Affiliations:** 1 Hoffmann Institute of Advanced Materials, Shenzhen Polytechnic, 7098 Liuxian Boulevard, Shenzhen 518055, China; 2 Department of Materials Science and Engineering, University of Sheffield, Sir Robert Hadfield Building, Mappin Street, Sheffield S1 3JD, UK; 3 School of Energy and Environmental Engineering, University of Science and Technology Beijing, Beijing 100083, China; 4 Chongqing Institute of Green and Intelligent Technology, Chinese Academy of Sciences, Chongqing 400714, China

**Keywords:** nuclear waste, nuclear industry, adsorption, membrane separation, combined processes

## Abstract

With the wide application of nuclear energy, the problem of radioactive pollution has attracted worldwide attention, and the research on the treatment of radioactive wastewater is imminent. How to treat radioactive wastewater deeply and efficiently has become the most critical issue in the development of nuclear energy technology. The radioactive wastewater produced after using nuclear technology has the characteristics of many kinds, high concentration, and large quantity. Therefore, it is of great significance to study the treatment technology of radioactive wastewater in reprocessing plants. The process flow and waste liquid types of the post-treatment plant are reviewed. The commonly used evaporation concentration, adsorption, precipitation, ion exchange, biotechnology, membrane separation, and photocatalysis are summarized. The basic principles and technological characteristics of them are introduced. The advantages and disadvantages of different single and combined processes are compared, and the development trend of future processing technology is prospected.

## 1. Introduction

Because of the non-renewability of fossil fuels and the limited availability of renewable energy, nuclear energy has received growing attention as an essential alternative energy source [1,2,3] With the development of new nuclear power plants worldwide, a large amount of radioactive waste, including wastewater, has been generated through various activities. Radioactive wastewater is generated during the operation of nuclear reactors and the application of radioisotopes in nuclear power plants. The chemical composition and the radioactivity level of the waste produced depend on the operation performed. Dissolved radionuclides are mobile in the natural environment. They can enter the aqueous environment, such as rivers and groundwater, if they are not adequately treated. This will inevitably increase the risk of human exposure to radionuclides [4,5,6]. Untreated radioactive wastewater discharged into the external environment will cause harm to human beings and nature [7]. The treatment of radioactive wastewater has always been the focus of social attention, and the key is to eliminate its threats to the environment and human health. In addition, the psychological burden brought by radioactivity to people is minimized.

**Figure 1:**
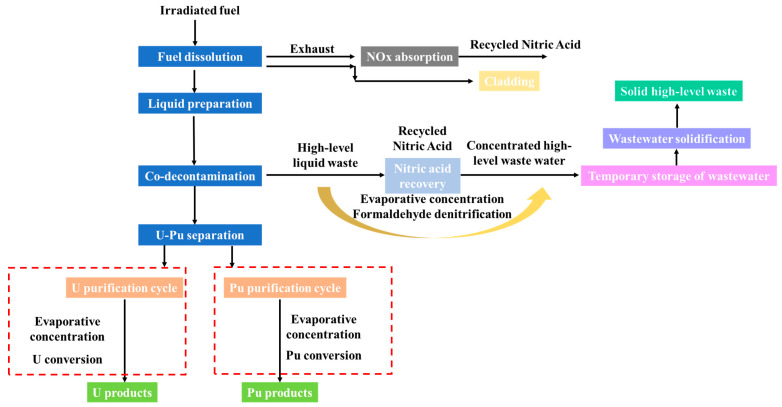
PUREX process flow chart.

To ensure the sustainable development of nuclear energy, it is necessary to reprocess spent fuels to recover useful nuclides and increase the utilization of uranium resources. On the other hand, it can reduce the volume of radioactive waste, the radioactivity, and the long-term toxicity of the radioactive waste [8,9]. Water reprocessing is a typical method for commercial nuclear fuels [10]. The U-Pu fuel cycle mainly relies on the classic PUREX process. It is a chemical process that uses tributyl phosphate to produce different extraction capabilities for uranium, plutonium, and other fission products to realize the separation and recovery of U and Pu products [11]. The simplified PUREX process is shown in Figure 1. The first is the dissolution of the fuel element. After the price adjustment, acid adjustment, and other processes to obtain the material to be extracted and separated, the co-extraction decontamination, uranium and plutonium separation, and back extraction are carried out. Then, the uranium and plutonium product solution are obtained, and finally the final solid product is obtained through purification and transformation.

In this process, generating a large amount of radioactive waste liquid is inevitable. Radioactive wastewater has the characteristics of complex composition, high radioactivity, high acidity, and high salinity, which requires further treatment and disposal. In addition, with the rapid development of nuclear technology, more radioactive waste is produced in different ways, such as the use of radioactive materials in hospitals, industry, or scientific research departments, mining of uranium ore, and the processing of thorium and uranium nuclear fuels [11]. The primary sources and characteristics of radioactive wastewater are shown in Table 1. The main nuclide in radioactive wastewater is uranium, and there are generally thorium and radium [12,13]. Compared with other pollutants, radioactive pollutants have an extremely long half-life (e.g., the half-life of uranium-238 is about 4.5 billion years), higher concealment, more muscular toxicity, and can exist stably for a long time. To prevent the radioactive waste generated during the use of nuclear technology from entering the environment, causing harm to the ecological environment system and human health, and at the same time to ensure the sustainable development of nuclear energy, it is necessary to pay special attention to the treatment and disposal of radioactive waste liquid. Therefore, how to treat nuclear wastewater efficiently and economically is a critical issue that needs to be resolved.

The wastewater treatment method is used to separate the pollutants contained in the wastewater, or convert them into harmless substances, so that the wastewater can be purified. There are four main categories: physical treatment, chemical treatment, physicochemical treatment, and biological treatment. Wastewater treatment technologies can be summarized into the following three categories: (1) Separation treatment separates pollutants from wastewater through the action of various forces. In general, the chemical nature of the contaminants is not altered during the separation process. (2) Conversion treatment changes the chemical nature of pollutants through chemical or biochemical effects. Pollutants are made into harmless substances or separable substances, and then separation treatment is carried out. (3) Dilution treatment can neither separate the pollutants nor change the chemical nature of the pollutants. Instead, by diluting and mixing, the concentration of pollutants is reduced to achieve the purpose of making them harmless.

In the treatment and disposal of radioactive wastewater, two principles are generally followed: one is to dilute and diffuse low-level radioactive wastewater and then discharge the diluted wastewater that meets the discharge standards with other waters; the other is to solidify the radioactive wastewater through concentration and solidification, followed by long-term isolation from the human environment and then letting it decay naturally. In comparison, the second principle is more widely applicable and can be used for high-, medium-, and low-level radioactive wastewater. In treating radioactive wastewater, obtaining a decontamination factor (DF) and a concentration factor (CF) is desirable. DF refers to the ratio of radioactivity concentration to mass concentration in influent and effluent water, and CF refers to the ratio of the original volume of the influent radioactive wastewater to the volume of the concentrated radioactive product after treatment. The DF indicates the reduction degree of the water’s radioactivity. A higher CF means a better volume reduction of the radioactive waste, which is conducive to further solidification and isolation.

Fundamentally, there are two main methods for handling radioactive aqueous substances: diffusion and storage. For low-level radioactive waste, most of the radioactive waste is transferred to a small-volume concentrate, and then the treated waste is diluted to allowable discharge concentration and discharged. High-concentration radioactive waste should be appropriately stored to isolate it from the environment. The treatment of radioactive wastewater is mainly aimed at radioactive metal elements, which can be chemically reacted to remove radioactive particles. The traditional treatment technologies include chemical precipitation [14], electrolysis [15], sulfide precipitation [16], and so on. Radioactive materials can also be separated and concentrated without changing their chemical form. Separation technologies mainly include adsorption [17,18], ion exchange [19], evaporation solidification [20], membrane separation [21], and so on. Technologies, such as flocculation, absorption, and enrichment via plant microorganisms [22], can also remove radioactive particles in water.

**Figure 2:**
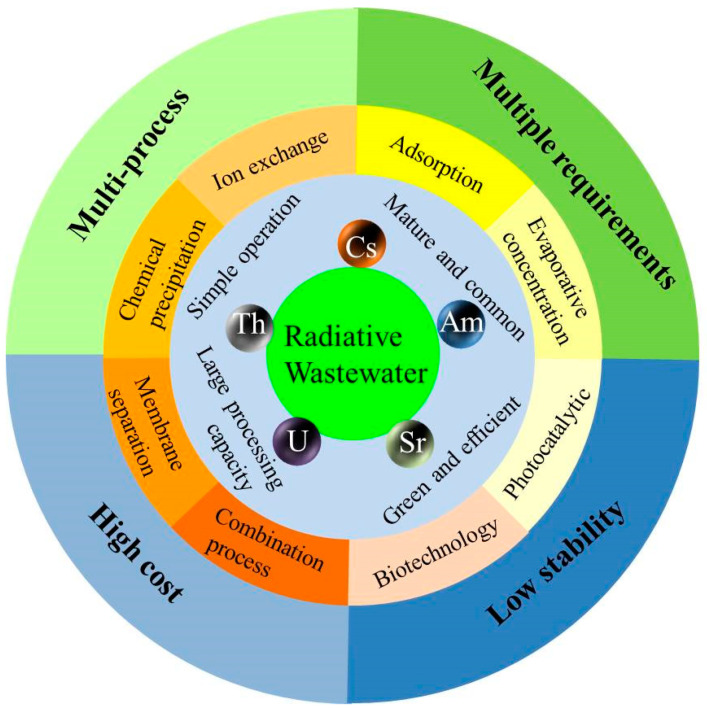
Structural framework classification diagram.

This review focuses on the treatment technologies of radioactive wastewater (as shown in Figure 2). It summarizes the mechanism and research progress of the traditional treatment process mentioned above but also supplements the novel photocatalytic treatment technology. By comparing different radioactive element treatment and recovery technologies, we look forward to the research direction of radioactive wastewater treatment.

## 2. Properties and Challenges of Radioactive Wastewater

Radioactive wastewater is produced in various ways and has different compositions. It not only affects the ecological environment but also has radiological hazards. The production of a large amount of radioactive wastewater will directly or indirectly affect human health and life. Therefore, developing efficient, fast, and economical radioactive wastewater treatment methods is imperative. In recent years, there have been many reports on the treatment technologies of radioactive wastewater, mainly including adsorption, precipitation, ion exchange, evaporation concentration, biotechnology, membrane separation, photocatalytic, etc.

In essence, the treatment of radioactive wastewater is to make radionuclides exist in a smaller volume of concentrates through a series of physical, chemical, and biotechnology processes, thereby reducing the concentration of nuclides in the radioactive wastewater and then through further treatment to make the wastewater meet the discharge or recycling standards. Currently, researchers’ research on radioactive wastewater mainly focuses on two aspects. On the one hand, it is to improve the existing process or optimize related parameters. Another aspect is the development of new materials. In actual working conditions, the composition of the waste liquid of the post-treatment plant is complex, the radioactivity is high, and the amount of wastewater is large. Therefore, when the treatment process is selected, if the waste liquid is to be thoroughly purified, future research should focus on consideration. Combining these technologies, combining processes, pretreatment of waste liquid, and optimizing operating conditions, such as evaporation concentration/flocculation precipitation and evaporation concentration ion exchange, to achieve the best treatment effect and reduce operating costs. Nevertheless, it is also necessary to solve the drawbacks brought about by the combined process.

The traditional radioactive wastewater treatment process is further optimized, and different treatment and disposal methods can be selected according to different objects to achieve the purpose of reduction, resource rationing, and harmlessness.

More safe and efficient membrane separation combination processes are actively developed, and a higher degree of automation control in the operation process is realized.

## 3. Treatment Technologies for Radioactive Wastewater

### 3.1. Ion Exchange

**Figure 3:**
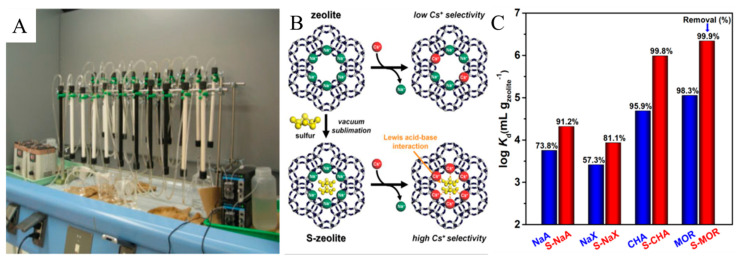
(**A**) Ion exchange process wastewater treatment device^24^; (**B**) Cs^+^ ion exchange selectivity enhanced by sulfur-encapsulated zeolite; (**C**) changes in distribution factor (K_d_) and removal efficiency after sulfur loading [32].

Ion exchange is a technology that uses the ions on the ion exchanger to exchange certain ions in the dilute solution to achieve the purpose of separating and extracting certain specific ions. As shown in Figure 3A, it is usually suitable for the treatment of waste liquids with low salt content [23]. In the post-treatment of radioactive waste liquid, the low-level radioactive waste liquid undergoes flocculation and sedimentation treatment. Since most particles and colloidal substances are removed after pretreatment, the remaining trace amounts of ionic nuclides in the solution are suitable for treatment with ion exchangers. According to the type of material, ion exchangers can be divided into two categories: resins and inorganic materials [24]. In early research, resin-based ion exchangers have received more attention. Bhattacharyya et al. studied the adsorption behavior of Th and U on the cation-exchange resin (Dowex50) through batch experiments and column operation experiments [25]. The results showed that Th has a stronger binding force to the resin than U. U can be eluted when the HNO_3_ concentration is in the range of 1 to 2 mol·L^−1^, while Th needs to be eluted at a higher HNO_3_ concentration (>6 mol). It shows that this resin can be used to separate U from Th according to the difference in elution acidity. Nur et al. [26] synthesized a resorcinol-formaldehyde polycondensation resin for the separation of Sr. The results showed that when the pH is 7.5–8.5, the ion exchange capacity for Sr is as high as 2.28 meq·g^−1^.

Although the use of resin-based ion exchangers has achieved good results, there are still some problems in using it to treat radioactive waste liquid, such as poor radiation resistance, heat resistance and chemical resistance, and high cost. In addition, the resin used to treat radioactive wastewater is usually not regenerated [27]. In comparison, inorganic ion exchangers seem more suitable for the treatment of radioactive wastewater, because they have higher chemical stability and radiation resistance and can generally provide higher exchange capacity and selectivity for various monovalent and divalent metal cations. Common inorganic ion exchangers include zeolite, titanosilicate, hexacyanoferrate metal oxides, and water-containing metal oxides, bentonite/clay, and ammonium phosphomolybdate (AMPs) [28,29,30,31], and so on. To improve the selectivity to Cs^+^, Han et al. [32] used vacuum sublimation to encapsulate the sulfur element inside the zeolite. Although the introduction of sulfur did not provide more adsorption sites, it provided its electronic part to the zeolite. Ions increase the ion exchange selectivity to Cs^+^ by providing additional interactions, as shown in Figure 3B,C. El-Naggar et al. [33] studied the adsorption of cesium (Cs^+^) in water by zeolite prepared from fly ash. The cation exchange capacity was 4.624 meq·g^−1^, and the maximum adsorption rate of Cs^+^ was 64.1%. Galambo et al. [34] used bentonite and montmorillonite to adsorb ^137^Cs in radioactive wastewater, and the maximum adsorption capacity was 0.88 mmol ^137^Cs·g^−1^. However, the adsorption or ion exchange performance of natural inorganic materials is relatively low. Therefore, natural inorganic materials are modified for radioactive wastewater treatment. Nerjee et al. [35] prepared a hexacyanoferrate (II) adsorbent (13X-CFC) by modifying zeolite by an in situ precipitation method and used this adsorbent for pilot tests. Under the conditions of a ^137^Cs concentration of 7 Bq·mL^−1^ and a flow rate of 0.3 Bq·mL^−1^, the adsorbent was used for a pilot test, which can treat more than 14,000 wastewater per resin bed volume. In addition, there are also reports of using modified clay to treat radioactive wastewater containing various concentrations of UO_2_^2+^ [36]. Traditional adsorbent materials have a slow adsorption rate, poor selectivity (such as clay and zeolite), small pore size (such as carbonaceous materials), poor regeneration performance (such as organic resin), and low adsorption capacity. Recent studies have shown that metal-modified nanocomposites and metal–organic framework materials have the advantages of high porosity, large specific surface area, and stable framework structure and can be used for the treatment of radioactive wastewater. Mobtaker et al. [37] prepared a cobalt hexacyanoferrate (CoHCNF)@polyaniline nanocomposite by chemical co-precipitation method, and the adsorption capacity for Cs^+^ at room temperature was 92.12 mg·g^−1^. The manganese dioxide-polyacrylonitrile (MnO_2_-PAN) composite material synthesized by Nilchi et al. [38] was used to remove ^137^Cs, and its adsorption capacity for I^−^ was 2.42 mmol·g^−1^. Yang et al. [39] prepared sodium hexacyanoferrate (NaCuHCF) functionalized magnetic nano-adsorbent for efficient magnetic removal of radioactive Cs^+^ from seawater. The Cs^+^ adsorption efficiency was 97.35% within 5 min, and the maximum adsorption capacity was 166.67 mg·g^−1^. In the presence of various competing ions such as Na^+^, K^+^, Mg^2+^, and Ca^2+^, this adsorbent can also selectively adsorb Cs^+^ efficiently, and the removal mechanism is ion exchange. In addition, the sodium hexacyanoferrate (NaCuHCF) functionalized magnetic nano-adsorbent still shows excellent Cs^+^ removal performance in seawater, with a removal efficiency of over 99.73%. In addition to physical and chemical adsorption and ion exchange, biosorbents prepared from natural organic materials have the advantages of low cost, stable chemical properties, and easy chemical modification. Genevois et al. [40] modified forestry waste with 2, 2,6,6-tetramethylpiperidine-1-oxyl radical (TEMPO) and nickel hexacyanoferrate (NiHCF) to prepare biosorbents for wastewater removal of Cs^+^. The results show that the maximum Cs^+^ adsorption capacity is 1.51 mmol·g^−1^. Similarly, the biosorbent prepared by Pangeni et al. [41] from persimmon waste also showed a fairly good adsorption capacity for Cs^+^ (0.76 mmol·g^−1^). It can be seen that the application of adsorption and ion exchange in the purification and treatment of radioactive wastewater has great potential. It should be noted that the ideal adsorbent or ion exchange material not only needs to have high adsorption or exchange capacity but also should have high stability and be easy to regenerate and reuse.

### 3.2. Chemical Precipitation

Chemical precipitation is a technology that reduces the specific activity of radioactive wastewater by co-precipitating the precipitant and the radionuclides in the waste liquid, thereby achieving the purpose of purification [42,43]. Because this method has the advantages of simple process, low cost, and wide application range, it was often used to treat radioactive wastewater in the early days. Commonly used precipitants include aluminum salts, phosphates, iron salts, soda, etc. Because most of the carbonate, phosphate, and hydroxide of radionuclides in wastewater are not easily soluble in water, they can be removed after precipitation. The pH of the solution, the stirring speed and length of time, and the amount of precipitating agent will all affect the precipitation effect. To enhance the coagulation effect, clay, active SiO_2_, polymer electrolyte, and other coagulants can be added [44,45]. Common precipitating agents have difficulty removing cesium, ruthenium, iodine, and other radionuclides at the same time, and some special precipitating agents or other methods are required. For example, cuprous chloride can be used to precipitate radioactive I^−^, which interacts with I^−^ to form a precipitate. Under the condition of a cuprous chloride concentration of 150 mg·L^−1^, the reaction only takes 15 min, and the removal rate of I^−^ with an initial concentration of 5.0 to 40.0 mg·L^−1^ is 95.8% [46]. However, traditional precipitants have difficulty removing ^137^Cs in the waste liquid. Rogers et al. [47] developed a new isotope dilution precipitation method to remove radioactive cesium from low-level wastewater by introducing non-radioactive ^133^Cs into the waste liquid. The increase of stable cesium is used to increase the total cesium concentration, and then sodium tetraphenylborate is used as the precipitating agent to achieve the purpose of removing a very small amount of ^137^Cs from the wastewater. The experimental results show that the final ^137^Cs activity can be reduced to the US Department of Energy standard 3.0 × 10^−6^ Ci·mL^−1^, which makes it possible for wastewater to be directly discharged into sewers or similar disposal methods. The process is not sensitive to pH and mixing time. However, when determining the initial dosage of precipitant, the influence of competitive ion potassium must be considered. The process is simple and direct and can be used as a treatment technology for low-level radioactive waste liquid containing cesium.

**Figure 4:**
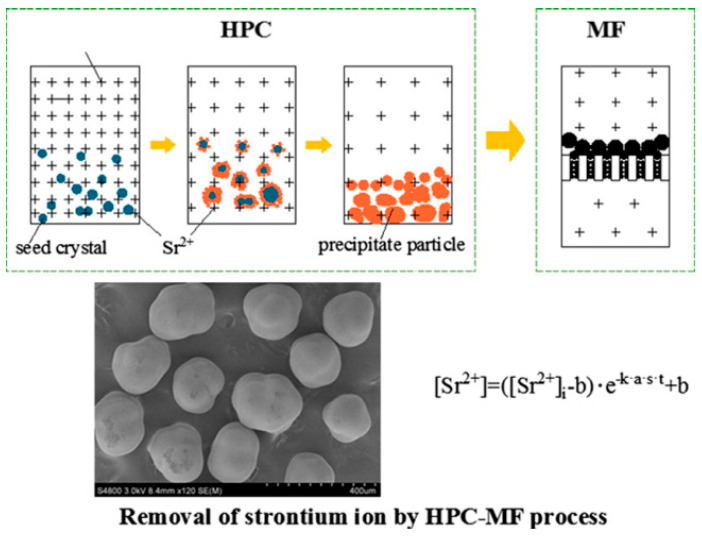
Sr^2+^ removal through the HPC-MF process [48].

Although the flocculation sedimentation method is simple and cost-effective to treat radioactive waste liquid, the difficulty of solid–liquid separation after use, the large amount of sludge, and the existence of secondary pollution limit the application of this technology. Based on this, Luo et al. [43] developed a co-precipitation microfiltration (PCM) process to treat strontium-containing wastewater. The results showed that the average decontamination factor for strontium was 577, and the concentration factor reached 1958, which solved the difficult situation of solid–liquid separation, indicating that the use of the PCM process has greater application prospects for the removal of strontium in the radioactive waste liquid. In addition, the hydraulic agitation co-precipitation microfiltration process (HPC-MF) proposed by Wu et al. [48] has a process flow for removing strontium as shown in Figure 4. When sodium carbonate, ferric chloride, and calcium carbonate are used as precipitants, flocculants, and seeds, the average and maximum decontamination factors are 842 and 1000, respectively, and the concentration factor (CF) is higher than 2650. The removal effect is further improved than the PCM process.

### 3.3. Membrane Separation

The membrane is a kind of functional material with selective separation, and its selectivity can achieve separation, purification, concentration, and other purposes [49]. According to different pore diameters, membranes can be divided into many types, such as reverse osmosis, ultrafiltration, nanofiltration, and microfiltration. Because of its advantages of saving energy, environmental protection, high efficiency, economy, and easy control, it is widely used in food, hydrometallurgy, energy, sewage treatment, and so on. Due to the unique structure and performance of the separation membrane, membrane technology has been widely used in water treatment. It was used in seawater desalination and pure water preparation in the early stage. Later, with the continuous development of technology, it has also been widely used and researched in radioactive waste liquid treatment. The membrane process used for treating radioactive waste liquid has the advantages of a high purification coefficient, large concentration volume, low energy consumption, simple system, flexible operation, and easy combination. It can be selected according to the composition of the radioactive waste liquid, the state of the solution, and the type of separation membrane [50,51].

**Figure 5:**
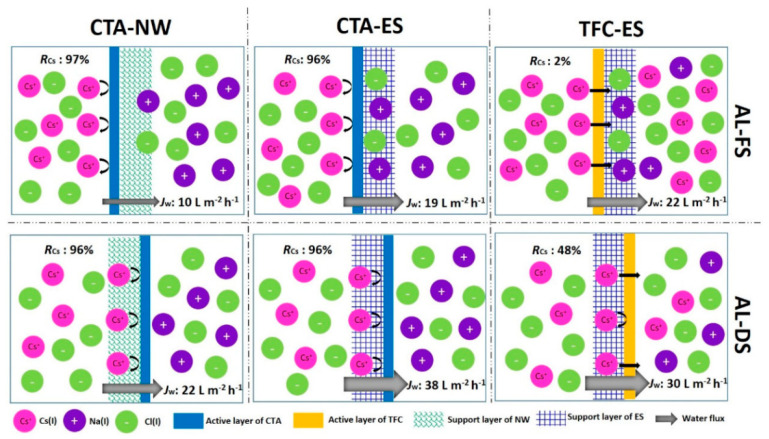
Cs(I) removal from radioactive wastewater by three FO membranes [58].

Microfiltration (MF) membranes can retain larger particles or macromolecules with a size of 0.1~1 μm. In nuclear technology, this process is usually used for pretreatment or filtration of large-particle precipitates produced in the concentrated liquid after precipitation. Under the action of pressure difference, particles with a particle size larger than the die hole size are intercepted to achieve a separation effect. Due to the large pore size, it is generally used to remove suspended solids in the waste liquid and other large particles and cannot directly and effectively remove the radioactive ions in the waste liquid. It usually needs to be used in combination with other processes. Zhao et al. [52] treated low-level wastewater containing plutonium by using a combination of flocculation sedimentation and microfiltration. By controlling the amount of ferrous sulfate and the pH of the solution, a plutonium removal rate greater than 99.9% can be achieved. In addition, the mixed waste liquid containing uranium, americium, and plutonium is processed. By using the combined process of flocculation and microfiltration, a single-stage total α removal effect of 99.87% is achieved. For the treatment of high-level radioactive waste, ceramic filters can be considered to achieve a higher decontamination coefficient and a higher concentration factor. The pore size of ultrafiltration (UF) membranes is generally 0.001~0.1 μm. Generally, only soluble compounds are allowed to pass, while colloids and various suspended solids are retained. In post-treatment, ultrafiltration technology is mainly used to remove colloids and suspended solids in the waste liquid. Ultrafiltration can be used as a pretreatment stage before reverse osmosis, and can also be combined with adsorption, precipitation, or complexation. Zhang et al. [53] studied the effect of low-concentration cationic surfactants on the removal rate of metal ions in the ultrafiltration process. The results show that when the amount of CTAB is lower than the critical micelle concentration, the removal rate of nuclide Cs^+^ increases from 24% ~33% to 50%. The removal rate of Sr^2+^, Co^2+^, and Ag^+^ is increased to more than 90%. The pore size of nanofiltration membranes is generally 1~2 nm, and most of them are composite membranes with electric charges. They are functional semi-permeable membranes that only allow certain low molecular weight solutes, low-valent ions, or solvent molecules to pass through. The retention effect of multivalent ions is higher than that of monovalent ions [54]. Lu et al. [55] prepared a TiO_2_-doped ZrO_2_ nanofiltration membrane and used it to treat simulated radioactive wastewater, achieving a rejection rate of 99.6% for Co^2+^, 99.2% for Sr^2+^, and 75.5% for Cs^+^, indicating that the nanofiltration membrane is effective for Co^2+^ and Sr^2+^ has a good removal effect. Reverse osmosis is an operation that uses differential pressure as the driving force to separate the solvent from the solution. It can trap various inorganic ions in the solution well, has a good concentration and purification effect on the solution, and is widely used in the treatment of radioactive waste liquid. Gu et al. [56] used a two-stage reverse osmosis device to investigate the treatment effect of boron-containing radioactive waste liquid. The results showed that the total salt removal rate was greater than 99.50%, and the total boron removal rate was greater than 84.30%. It has a good effect on both ^137^Cs and ^90^Sr in wastewater. The removal effect proves that the reverse osmosis method has a good purification effect on the radioactive waste liquid. In addition to the typical membrane separation techniques described above, electrodialysis, membrane distillation, supported liquid membranes, etc., have also been extensively studied in the field of radiochemical separation [57,58]. Liu et al. [58] used a NaCl solution and simulated seawater as the extraction solution to remove Cs(I) from radioactive wastewater through three forward osmosis (FO) membranes, as shown in Figure 5. Compared with other membrane separation processes, FO has a higher removal efficiency of Cs(I). The CTA (cellulose triacetate) membrane achieves a high Cs(I) retention rate of 90.35%–97.15%.

Although membrane separation technology has certain advantages and shows great potential with more environmental protection advantages, it should be considered in practice that membrane fouling is still a severe problem for maintaining membrane flux and reducing system maintenance frequency. In addition, for the unique environmental system of radioactive waste liquid, higher radioactive exposure will inevitably destroy the surface structure of the membrane, resulting in a decrease in membrane performance and a shortened lifespan [59]. The ability of the currently used membrane materials to withstand harsh environments needs to be further explored. Under relatively high levels of radioactivity, the surface structure of the membrane will inevitably be destroyed, resulting in impaired performance and shortened life. Therefore, to overcome the above problems and promote the better development of radioactive wastewater treatment, on the one hand, we can consider optimizing process parameters, improving the process flow, and reducing the contact time; on the other hand, we can consider the research and development of anti-fouling membranes, ceramic membranes, etc. In addition, in the actual radioactive wastewater treatment, membrane technology is limited by the requirement for rapid removal of nuclides. As subsequent solid–liquid separation units, MF and UF must be combined with precipitation, adsorption, flocculation, and other methods. Although NF and RO can directly intercept radioactive ions in water bodies, it is necessary to judge whether a pretreatment process is required according to the water quality. Currently, some water plants in the United States and Canada have tried applying membrane technology to actual radioactive wastewater treatment. However, it is still necessary to develop new membrane materials and membrane technologies to treat radioactive wastewater to make this technology more efficient.

### 3.4. Evaporative Concentration

For the treatment and disposal of radioactive waste liquid, evaporation technology is commonly used to concentrate it [60]. The basic working principle is to send the radioactive waste liquid into the evaporator and heat it with an electric heater or introduce heating steam. The water in the waste liquid is heated to evaporate to form water vapor, which is then cooled by the condensation system to form condensed water. After passing the test, it is discharged or reused, while the non-volatile radionuclides remain in the water, are concentrated and discharged, and then undergo subsequent solidification treatment [61]. Evaporative concentration is a proven method that can significantly reduce the amount of radioactive wastewater [62]. It has been widely used in treating radioactive waste liquid, especially for wastes containing relatively high concentrations and hardly any volatile radionuclides. It has a purification coefficient and the advantages of a high-volume reduction effect, great flexibility, wide application range, and the ability to be combined with various technologies. At the same time, this method does not require additives and will not cause secondary pollution [63].

**Figure 6:**
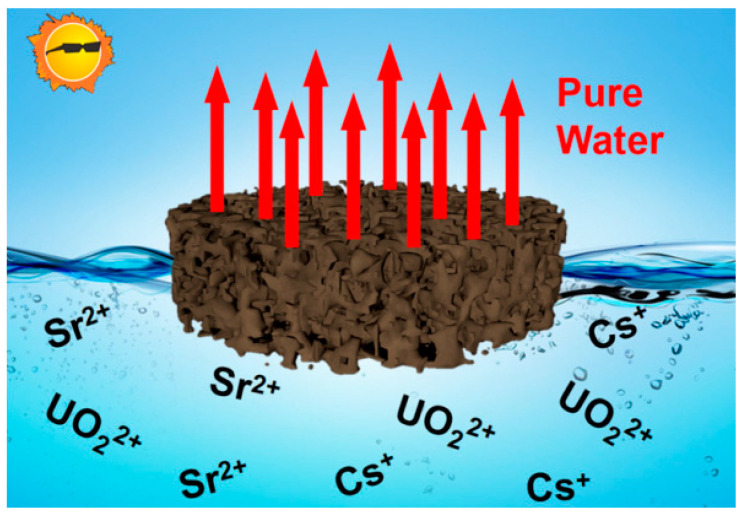
Efficient solar-driven evaporative radioactive wastewater treatment [68].

To improve evaporation efficiency and reduce equipment operating costs, researchers have spared no effort in the development of new evaporators and have achieved remarkable results in the development of various evaporators. Based on the performance comparison between an externally heated evaporator and a kettle-type evaporator, Hu and Lu et al. [64] proposed the use of a kettle-type evaporator to treat the high-level liquid waste produced by the spent fuel reprocessing plant in their country. It has unique advantages in the treatment of acidic radioactive waste liquid, such as the easy realization of the “continuous evaporation-denitration” process. Aiming at a certain amount of gas produced in the denitration process, the design of the kettle evaporator system structure can also solve the foaming phenomenon during denitration and reduce the radioactivity of the condensate. Given the production capacity being affected due to the limited heat exchange area, measures have also been proposed to appropriately increase the internal heating exchange pipes and stirring equipment to increase the heat exchange area and improve the heat exchange capacity. In the traditional evaporation and concentration process of radioactive wastewater, the kettle-type, rising-film-type, and natural-circulation-type evaporators are more used [65]. However, the direct heating method during use will lead to the consumption of a large amount of primary steam or electric energy, which consumes high energy. At the same time, the consumption of condensate is also large. Compared with traditional evaporation, MVR (mechanical vapor recompression) technology realizes energy saving based on the principle of the heat pump. The condensate is directly used to preheat the raw material liquid, eliminating the additional supply of condensate [66] (Xia et al., 2019). Xu et al. (2016) used a set of 50 L·h^−1^ MVR evaporation devices to carry out a simulated wastewater evaporation experiment containing strontium, cesium, and cobalt nuclides. The results show that the average decontamination factor of the device can reach more than 7 × 10^5^, and the energy saving is as high as 88.7% compared with the traditional evaporator, which proves that the MVR device has great potential in the purification of radioactive sewage. In addition, Wei and Fang et al. [67] developed a vacuum evaporation and concentration device to treat radioactive wastewater generated by special military tasks. It mainly uses the lower boiling point of vacuum-state water to achieve the effect of impurity removal and purification through simple vacuum distillation. Thermal test results show that the total α and β purification coefficients for low-level radioactive waste liquid reach 3.14 × 10^4^ and 2.49 × 10^4^, respectively, and the total α and β purification coefficients for intermediate-level liquid waste reach 4.37 × 10^4^ and 2.04 × 10^6^, respectively. The equipment is operating stably. The effluent quality meets the requirements and meets the relevant discharge standards. In addition to artificial heat sources, heating from solar energy is also widely considered. Yu et al. [68] designed a monolithic sponge with a three-dimensional porous structure as a solar evaporator, as shown in Figure 6. Under a single sunlight exposure, the sponge has good absorption, light and heat, heat insulation, and fast water transmission characteristics, so it achieves a fast evaporation rate (1.60 kg m^−2^ h^−1^) and a high interfacial water evaporation efficiency (92%). Solar-driven interface evaporation can effectively treat radioactive wastewater and enrich various radionuclides in a more energy-efficient way.

In addition, the amount of radioactive waste liquid produced in hospitals, scientific research units, and other places is usually relatively small, large-scale evaporation devices are used, and equipment investment and construction costs are relatively high. Here, infrared heaters have many applications. The basic principle of infrared heaters to evaporate liquids is that water molecules have good infrared absorption performance [69]. Xu and Yao et al. [70] used the infrared heating and evaporation method to treat radioactive wastewater with a purification coefficient of 104. Compared with the traditional evaporation method, this method only evaporates the surface water without boiling and foaming, and the purification coefficient is higher; in addition, the equipment is safe and reliable, easy to operate, not easy to corrode, and has a lower cost. It is a unit that produces a small amount of waste liquid. The evaporation method technology is relatively mature and a viable choice for treating small-volume and high-level radioactive wastewater. Generally speaking, since most radionuclides are not volatile in water bodies, the radioactive wastewater can be evaporated and concentrated to gradually vaporize the water in the wastewater into water vapor, which is then cooled to form condensed water. Most of the radionuclides are kept in the vaporized residual liquid, and then the concentrated liquid is solidified and isolated to obtain a higher DF. However, the evaporation method has limitations for removing volatile nuclides in water. For example, iodide in radioactive wastewater is very easy to volatilize, so the treatment of wastewater containing radioactive iodine nuclides is not suitable for evaporation. The evaporation method has shortcomings: it consumes energy and low heat.

The evaporation method has the following shortcomings: it consumes a lot of heat energy, has low heat utilization, and is expensive; it is not suitable for processing waste liquids that easily foam and contain volatile nuclides (such as iodine, krypton, etc.); when processing acidic high-level waste liquids, the boiling point increases, the efficiency decreases, and equipment corrosion increases as the acid concentration increases; in addition, the appearance of fouling, explosion, etc., should also be considered during design operation [71,72]. Therefore, further development of new high-efficiency evaporators and exploration of new evaporation technologies will be of great significance to the progress of this technology.

### 3.5. Adsorption

**Figure 7:**
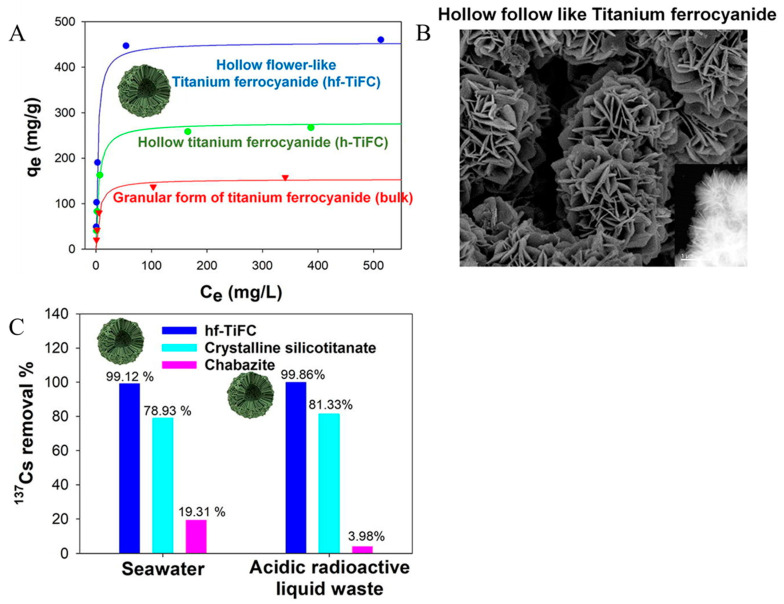
Hollow flower-shaped titanium ferrocyanide (hf-TiFC) composed of two-dimensional TiFC flakes is used to remove ^137^Cs from water and enhance the adsorption performance of Cs: (**A**) isotherms and the influence of pH on the Cs adsorption performance; (**B**) SEM images; (**C**) removal effect [77].

The use of adsorption technology to treat radioactive waste liquid generally refers to a technical means of using porous adsorbent materials to remove radionuclides in the waste liquid. Different types of adsorbents can be selected for the treatment depending on the nature of the waste liquid. The different types of adsorbent materials can be roughly divided into inorganic adsorption materials (mainly zeolite, activated carbon, bentonite, etc.), biomass adsorption materials (such as cellulose, chitosan, etc.), and synthetic polymer materials (such as resins) [73,74,75]. As far as inorganic adsorbent materials are concerned, zeolite is cheap and easy to obtain and has a higher decontamination coefficient for radionuclides in water, between 62 and 68. It is about ten times or even 20 times higher than other materials and has the functions of ion exchange and filtration [76]. Although activated carbon has strong adsorption capacity and good decontamination and impurity removal, its poor regeneration performance and high cost limit its application. It should be recognized that natural materials generally do not have high adsorption capacity. Therefore, more energy should be focused on developing adsorbent materials with high adsorption capacity, high selectivity, and good reproducibility. Yang et al. [77] synthesized a hollow flower-shaped titanium ferrocyanide (hf-TiFC) (as shown in Figure 7 by controlling the acidity), which was combined with conventional Cs adsorbents (such as zeolite and crystalline titanate silicate (CST)). Compared with Cs, the adsorption performance of Cs is significantly enhanced. Compared with two-dimensional TiFC, due to the increase of the effective surface area of hf-TiFC, the maximum adsorption capacity (454.54 mg·g^−1^) is significantly increased, which is three times higher than that of two-dimensional TiFC. In the radioactivity test, even a low-concentration hf-TiFC (0.1 g·L^−1^) showed excellent removal performance in simulated seawater and nuclear waste liquid at pH = 1 and 5.7 M Na^+^, at the initial ^137^Cs. When the specific activity is about 110 Bq·g^−1^, the removal efficiency exceeds 99.1%. Since strontium has a long half-life, the removal of strontium is essential for radioactive waste management. Eka et al. [78] synthesized a melamine-styrene-based polymer (MSBP) with good radiation resistance, which was used to remove Sr^2+^ ions from the solution. The effects of pH value, adsorbent dosage, initial concentration of Sr^2+^, contact time, temperature, particle size, etc., on the adsorption were investigated. The results showed that the maximum adsorption capacity of MSBP adsorbent for Sr^2+^ can reach 142.9 mg·g^−1^. Yang et al. [77] synthesized copper-sodium ferricyanide (NaCuHCF) functionalized magnetic nano-adsorbent to remove radioactive cesium from seawater. The results show that the NaCuHCF-PEI-MNC adsorbent can achieve 97.35% Cs adsorption within 5 min, and the maximum adsorption capacity for Cs can reach 166.67 mg·g^−1^. The adsorbent has good selectivity and stability. It can stably exist in the pH range of 4~10. It can also selectively adsorb Cs^+^ in the presence of competing ions such as Na^+^, K^+^, Mg^2+^, and Ca^2+^. Experiments with real seawater showed excellent removal performance for Cs+, with a removal rate of over 99.73% and a purification coefficient of over 372.

From the above point of view, there is much room for applying adsorbents in radioactive waste liquid treatment. However, the components of the radioactive waste liquid system are complex, and the effectiveness of the adsorbent under some harsh conditions still needs further study. It should be realized that the ideal adsorbent should have high adsorption capacity and high selectivity. It can maintain stability under various environmental conditions, is easy to regenerate, and can be reused.

### 3.6. Biotechnology

**Figure 8:**
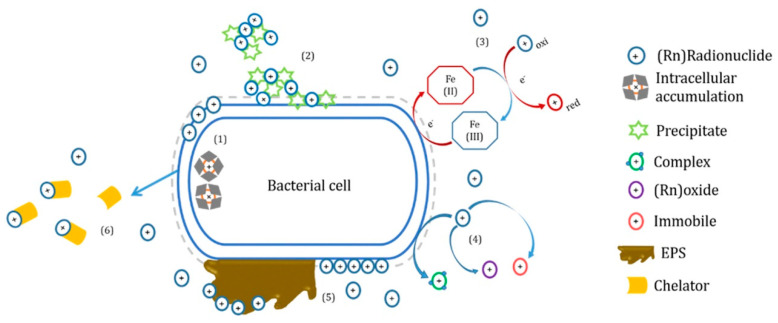
The mechanism of biological treatment of low-level radioactive waste liquid [79].

Biotechnology removes radionuclides through biotransformation, biosorption, bioaccumulation, sedimentation, and solubilization mechanisms using plants or microbial cells as media [79]. As shown in Figure 8, this technology has the advantages of environmental protection, high efficiency, mildness, low cost, low energy consumption, and no secondary pollution. It significantly reduces radioactive waste [80].

Biotechnology has been studied for the treatment of low-level radioactive waste liquid since the 1960s, and great progress has been made at present [81]. Ferreira et al. [82] and others cultivated bacterial colonies in uranium mining areas and non-uranium mining areas to treat radioactive waste liquid. It was found that the colonies cultured in uranium-bearing mining areas had better radioactive organic waste liquid degradation and radionuclide adsorption capabilities than those cultured in non-uranium mining areas. Among them, at higher concentrations, the colonies cultured in the soil of uranium-bearing mining areas can adsorb 92% of uranium and 100% of ^241^Am and ^137^Cs. The above research results indicate that the colonies cultivated in the soil of uranium-bearing mining areas are very suitable for processing large-volume radioactive organic waste liquid. Gorbunova et al. [83] used microbial colonies to pretreat the low-level radioactive organic waste liquid. The results showed that due to the presence of active substances on the biological surface, the microbial colonies can oxidize 60% of the organic components into water and carbon dioxide, which can effectively reduce the volume of radioactive waste liquid. The process of using microorganisms to treat radioactive waste liquid is relatively complicated and is greatly affected by environmental factors such as pH, type of nuclide, treatment time, and initial concentration. In a study by Liu et al. [84], it was first proposed to use Bacillus subtilis to treat Sr^2+^ in low-level radioactive waste, and the effects of pH, temperature, and initial ion concentration on the adsorption effect were investigated. It was found that when pH = 6.3, temperature is 20 °C, initial concentration is 15 mg·L^−1^, and adsorption time is 24 h, the removal rate can be as high as 96.3%. Tsezos and Volesky et al. [85] screened some waste microorganisms produced during industrial fermentation for the treatment of radioactive waste liquid containing thorium and uranium metal ions. The results show that when pH = 4, the maximum adsorption capacity of Rhizopus for thorium and uranium is greater than 180 mg·g^−1^, and the removal rate for uranium is 2.5 and 3.3 times that of ion exchange resin and activated carbon, respectively. The removal rate of thorium is 20 and 2.3 times that of ion-exchange resin and activated carbon under the same conditions, respectively. The biosorption of radionuclides such as Th, U, Sr, and Cs by different types of biosorbents has been widely reported [83]. Ahmadpour et al. [86] and others used almond shells, eggplant peels, and moss as biosorbents to treat the radionuclide strontium in water. It is found that the type of material, the pretreatment method, the amount of the initial adsorbent, and the concentration of metal ions in the initial solution all have a significant impact on the adsorption effect. Through comparison of batch adsorption experiments, at 25 °C, almond shells can achieve a 96% removal rate of Sr^2+^ in 2 min, and the maximum adsorption capacity can reach 116.3 mg·g^−1^.

The use of biotechnology can not only adsorb radionuclides but also reduce and recover uranyl ions through the intervention of bacteria and other microorganisms [87]. This technology can also be used for other radionuclides and some precious metals. However, the cell damage caused by radiation doses beyond a specific range should also be considered during use.

### 3.7. Photocatalysis

**Figure 9:**
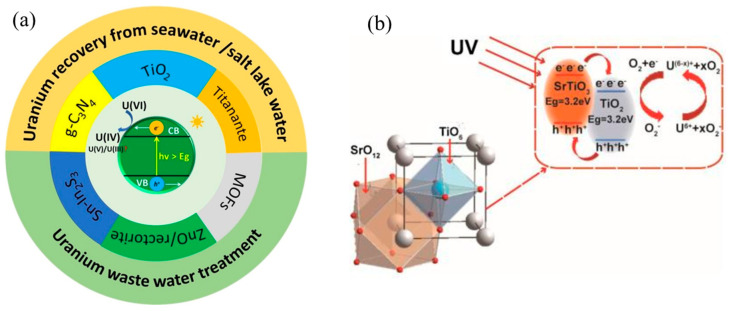
Common types of photocatalysis (**a**) and TiO_2_ catalytic mechanism (**b**) [94].

Photocatalysis refers to converting solar energy into chemical energy in the presence of a photocatalyst. In this process, the photocatalyst can chemically change the reactant after absorbing light, and the excited photocatalyst can interact with the reactant many times. Intermediate substances are produced while ensuring that it remains unchanged before and after the reaction. When the incident light’s quantum energy is equal to or greater than the forbidden bandwidth of the semiconductor, the valence band electrons are excited to transition to the conduction band, correspondingly generating holes h+vb in the valence and forming photogenerated electrons e-CB in the conduction band. The photogenerated electrons formed on the conduction band have reduced reliable power, while the photogenerated holes on the valence band have solid oxidizing power. They can migrate to the semiconductor surface and undergo corresponding redox reactions with the contaminants adsorbed on the surface. In the radioactive waste liquid, organic waste liquid and tritium-containing waste liquid are two special waste liquids. They cannot be processed by evaporation, concentration, ion exchange, and membrane separation and require exceptional management. Mainly include radioactive waste oil, organic solvent, waste organic scintillation fluid, and decontamination fluid. Photocatalytic treatment technology can be used for reference in treating organic wastewater in other fields. The generated photogenerated electrons are mainly transported to the surface of the semiconductor by transferring electrons and holes in the following forms. Combining electrons and holes at the impurity or defect in the semiconductor, the recombined electrons and the acceptable electron contaminants (acceptors) adsorbed on the semiconductor surface undergo a reduction reaction. The holes are transported to the surface to undergo an oxidation reaction with the donors (donators). The common types and mechanisms of photocatalysis are shown in Figure 9a,b. In the photocatalysis process, it is essential to accelerate the separation of electron–hole pairs, reduce the rate of electron–hole recombination, and improve the efficiency of photocatalysis.

**Figure 10:**
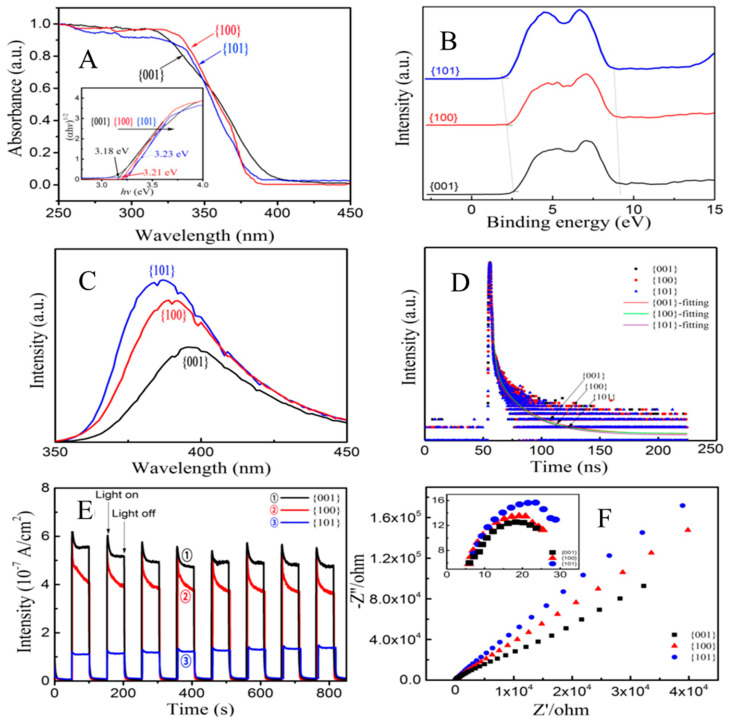
Performance analysis of {001}, {100}, and {101} crystal planes of TiO_2_: (**A**) UV–Vis diffuse reflectance spectrum; (**B**) XPS spectrum of the valence band. (**C**) Stable fluorescence emission spectrum. (**D**) Time-resolved PL spectrum. (**E**) Transient photocurrent. (**F**) EIS curve [93].

Take the common nuclide uranium (U) as an example. Uranium has a variety of valence states, including U(VI), U(V), U(IV), and U(III), where U(VI) and U(IV) are two forms of U that can exist stably in the environment. U(VI) is highly soluble, highly toxic, and easily migrates in the environment, while U(IV) is a poorly soluble substance with low toxicity. Reducing the easily soluble and highly toxic U(VI) to the insoluble and low toxicity U(IV) is one of the most ideal ways to separate and recover U from wastewater or fix it for a long time. Salomone et al. [88] found that the reduction efficiency of uranyl acetate in acetic acid (16%) is higher than that of uranyl nitrate in nitric acid (4%). However, in the presence of 2-PrOH and the case of a quartz photoreactor, the uranyl nitrate in nitric acid is reduced by 98% within 60 min. The efficiency is much higher than that of perchlorate in uranyl perchlorate and uranyl acetate in acetic acid. Wang et al. [89] found that sodium formate can increase the adsorption of U(VI) on the surface of TiO_2_, and the maximum adsorption capacity of U(VI) in the presence of sodium formate increased to 44 mg·g^−1^. The photocatalytic reduction rate constant increased 17 times. Feng et al. [90] synthesized Sn-doped ln_2_S_3_, which has a high specific area. When the optimal ratio Sn:ln = 1:4.8, the reduction efficiency in 40 min reaches 95%, which is about 15.6 times faster than pure ln_2_S_3_. Lu et al. [91] synthesized boron-doped g-C_3_N_4_. Among them, 1.0%wt of B doping has the best effect, which can completely reduce 200 mL, 0.12 mM U(VI) within 20 min. In addition, the efficiency of five cycles is greater than 90%. Guo et al. [92] synthesized zinc oxide/retorite composite material by sol–gel method. Using methanol as a sacrificial organic matter improves the adsorption capacity and photocatalytic reduction activity of U(VI) on zinc oxide/retorite composites. In addition, the composite material still shows high light reduction activity after four reaction cycles under visible light irradiation. Chen et al. [93] prepared anatase TiO_2_ with {001}, {100}, and {101} planes, respectively, studied its effectiveness in the removal of U(VI), and calculated and studied it by density functional theory (DFT) surface chemistry at the molecular level. According to Figure 10, the experimental results of TiO_2_ show that compared with {100} and {101} plane TiO_2_, {001} plane TiO_2_ has the best adsorption capacity and photoreduction ability. The DFT calculation results show that the adsorption of U(VI) on the three surfaces leads to the formation of inner spherical composites. Among them, monodentate composites are most suitable for {001} plane TiO_2_, and bidentate complexes are most suitable for {100} and {101} plane TiO_2_.

In the research field of photocatalytic reduction of uranium, there are many types of photocatalysts, including TiO_2_ and its related complexes [89,95,96], iron oxide and its complexes [97,98], g-C_3_N_4_ and its complexes [99,100], and other photocatalytic materials. However, most photocatalytic materials have low charge separation efficiency and a low utilization rate of sunlight, which makes this method yet to be applied to the treatment of natural uranium-containing wastewater. Therefore, developing a new visual light catalytic reduction system is an important research direction for the photocatalytic treatment of uranium-containing radioactive wastewater.

## 4. Comparison of Different Radioactive Waste Treatment Technologies

**Figure 11:**
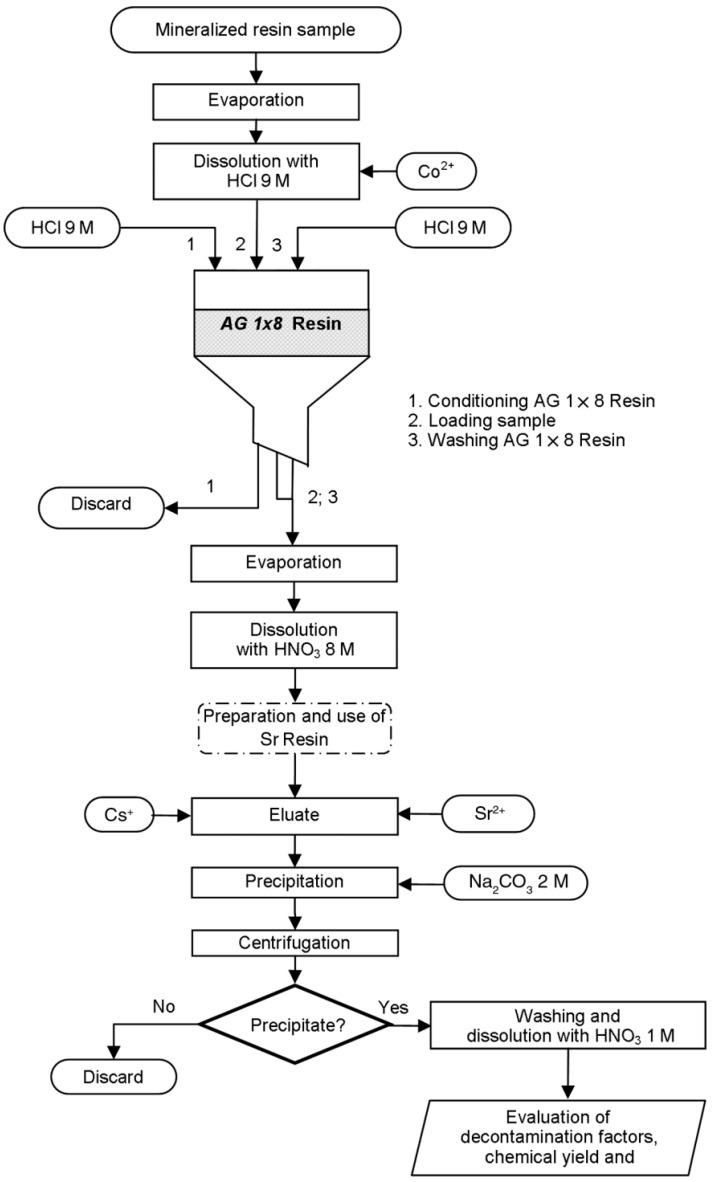
Flow chart of separation using ion exchange chromatography, extraction chromatography, and precipitation [101].

Many technologies, such as flocculation and precipitation, ion exchange, evaporation and concentration, solvent extraction, and membrane separation, have been widely used in treating and disposing of radioactive wastewater. Various technical advantages and disadvantages are shown in Table 2. It can be seen from the table that each technology has its characteristics and limitations, but the ultimate goal is to reduce the generation of radioactive waste. Reduce potential environmental hazards, recover valuable materials, and minimize the volume of radioactive waste liquid to ensure the sustainable development of nuclear technology utilization. The use of flocculation and sedimentation, membrane separation, and ion exchange or adsorption technologies may have deficiencies such as narrow application range, poor versatility, high-performance requirements for materials, and secondary pollution. Whether it is flocculation sedimentation, ion exchange, membrane separation, or adsorption technologies, the treated waste liquid meets the emission standards and can be directly discharged or recycled. Then, evaporation and concentration treatment are carried out to minimize the volume of waste liquid. The curing process is then carried out. Therefore, combining multiple treatment processes can develop their advantages and compensate for their shortcomings. As shown in Figure 11, by combining ion exchange chromatography, extraction chromatography, and precipitation, the activity and recovery rate of the ^90^Sr purified fraction has been well improved. However, the combined process also has certain drawbacks. It has high design requirements and high operating costs. Therefore, both application occasions and operation and maintenance are restricted.

## 5. Integrated Treatment of Radioactive Wastewater

In recent years, wastewater treatment equipment has developed towards miniaturization, and integrated wastewater treatment equipment has emerged as the times require. At present, integrated wastewater treatment equipment has been widely used in urban domestic water treatment and industrial water treatment in Europe, America, Japan, and other countries and regions. Because of its low investment, convenient operation and management, and low cost, it eases the financial pressure on water treatment equipment in the water treatment industry. Integrated radioactive wastewater treatment has gradually become a new research hotspot in water treatment.

Integrated wastewater treatment equipment can basically meet the wastewater treatment requirements of living quarters and small- and medium-sized enterprises. It has the advantages of less investment, quick results, simple operation, and no special training for operators. For the treatment of radioactive wastewater, its advantages are quite prominent.

Large sewage treatment plants or workshops need to occupy a large amount of construction area, which increases the burden on enterprises. Integrated equipment does not need a lot of land. Many devices can be buried underground, which saves space and does not cause landscape damage to living areas or scenic spots.

With the gradual increase of domestic and industrial water use, the scarcity of water resources is a major problem that human beings must face. The untreated radioactive wastewater is directly discharged into nature, causing serious environmental pollution. Most of the treated radioactive wastewater can be reused, saving water resources. Since the integrated equipment does not require a large-scale pipeline layout, it can arrange water reuse nodes more flexibly, which is more advantageous than large-scale traditional water treatment equipment.

Integrated radioactive wastewater treatment equipment realizes the integration of radioactive wastewater treatment technology and integrates the original single technology into one device. As the country and enterprises gradually increase the requirements for radioactive wastewater treatment, the integration of integrated equipment will become higher and higher, which will promote the progress of wastewater treatment technology.

## 6. Concluding Remarks and Perspectives

In this sense, evaporation and concentration technology has a broader range of applications in the field of radioactive waste treatment because of its high decontamination coefficient, significant concentration multiple, and robust versatility. It can be combined with a variety of technologies. Evaporative concentration also plays a crucial role in the post-treatment of spent fuel in the water process, mainly used to minimize the volume of radioactive waste liquid, recycle nitric acid, and increase the concentration of metal ions. According to the existing experience, when using evaporative concentration, there are still the following shortcomings: First, the energy consumption is large, the heat utilization rate is low when the acidic waste liquid is processed, the boiling point rises as the solution concentration increases, and the equipment corrodes seriously. New technologies can be considered to improve evaporation efficiency and reduce energy consumption. When processing the raffinate produced by the solvent extraction process after spent fuel reprocessing, due to the incomplete phase separation and the solubility of TBP in the water phase, a certain amount of organic phase will be entrained in the water phase. In the process of evaporating and concentrating the raffinate, TBP and its degradation products will be complex with nitric acid or heavy metal nitrates (uranyl nitrate and plutonium nitrate) to form complex nitroso compounds, namely “red oil.” At a specific temperature, there may be a risk of a “red oil” explosion. This requires strict temperature monitoring and reduction of phase entrainment to reduce risk [102]. Evaporation and concentration technology are significant in treating radioactive waste liquid because of their high purification coefficient, flexibility, and versatility.

However, there are still some shortcomings, such as the risk of “red oil” explosion, corrosion of equipment, etc. Therefore, removal of entrainment from the "source" can be considered. The interface evaporation technology is adopted to improve the deficiencies in the evaporation process. To better realize the environmentally friendly and sustainable development of nuclear energy and nuclear technology applications. Traditional technologies have more or fewer limitations: high cost, secondary pollution, etc. For example, chemical precipitation is relatively simple and cost-effective. However, this technology often fails to reduce uranium concentration below the legal limit and tends to produce secondary pollutants. Membrane separation technology equipment is prone to blockage due to long-term use, and equipment maintenance costs are high. Solvent extraction will lead to producing organic waste solvents and so on. These factors are inevitable.

Therefore, low-cost strategies for efficient nuclear waste removal and minimization are essential. Catalytic technology is considered ideal for waste treatment due to its advantages of environmental protection, non-toxicity, low cost, stable performance, and no secondary pollution. It also conforms to the IAEA’s design principles for nuclear waste treatment and disposal minimization. The birth of photocatalysis opened up a new world for treating nuclear waste. Photocatalytic treatment technology can be used for reference in treating organic wastewater in other fields, especially macromolecular organic waste liquids such as printing and dyeing and pesticides. The application of photocatalysis to actual domestic sewage treatment is still under study. The nuclear industry wastewater treatment application is still in its infancy, so relevant research and data must be supplemented.

With the development and utilization of nuclear energy technology, the treatment of nuclear wastewater has attracted increasing attention. In the treatment of radioactive wastewater, the chemical precipitation method has the advantages of a simple process, low cost, and broad applicability; however, the DF is low, and concentration is complex, and the separation exchange/adsorption method has a high decontamination factor and concentration multiplier, and its selectivity is poor. Membrane technology can make up for the defects of the above methods. However, the membrane technology requires high raw water quality, and the biological method is still in the research state and cannot be carried out for large-scale practical application. Radioactive wastewater treatment should consider high treatment efficiency, sludge concentration, cost, and safe and reliable performance. The combination process of multiple methods will be the crucial future development trend of radioactive wastewater treatment.

## Figures and Tables

**Table 1:** Main sources of radioactive wastewater.

Sources	Typical Radioisotope	Characteristics
Nuclear Research Center/Radioisotope Laboratory	According to the target’s yield and purity, there are many varieties, short-term active nuclides, and long-term radionuclide mixure.	a. After the ion-exchange resin is regenerated, the batches whose pH value is close to neutral are generally more uniform; b. Small size, high specific activity, high chemical concentration;
Nuclear Power Plant	^3^H, ^14^C, U (^233^U, ^234^U, ^235^U, ^238^U) and Th (^228Th^, ^232^Th), etc.	a. The volume may be large, and the chemical composition is uncertain; b. Very high specific activity and chemical concentration;
Scientific research	Variable, short-lived, and long-lived radioisotopes	Extremely variable inactivity, volume, chemical concentration, etc.;
Radiolabels and radiopharmaceuticals/medical diagnosis and treatment	^14^C, ^3^H, ^32^P, ^35^S, 1^25^I, ^99^Tcm, 1^31^I, ^85^Sr	a. Predictable small volume of chemical composition; b. Mainly comes from the patient’s large amount of urine, and a small amount comes from the preparation and processing process;
Rare earth metal mine beneficiation wastewater	It varies greatly depending on the type of ore	a. Large size and uncertain chemical composition; b. Often mixed with other toxic heavy metals;
Industrial and pilot plants	Depends on the application, for example in the instrument industry (^226^Ra, ^147^Pm)	The volume may be large, and the chemical composition is uncertain.

**Table 2:** Comparison of advantages and disadvantages of different radioactive waste liquid treatment technologies.

Processing Technology	Advantages	Disadvantages
Ion exchange	High selectivity and simple operation	Affected by salinity, regeneration is difficult, secondary waste is generated
Chemical precipitation	Suitable for processing large volumes of high-concentration waste liquid, simple, convenient, and low-cost	Difficulty in solid–liquid separation, poor treatment of anionic radionuclides
Evaporative concentration	High decontamination coefficient, large concentration ratio, mature method, strong versatility, and great flexibility	High energy consumption, low heat utilization rate, equipment corrosion, and scaling
Membrane separation	Large processing capacity, flexibility, easy to be combined with multiple methods	High cost, easy to pollute the membrane, poor radiation stability
Biotechnology	Environmentally friendly, no secondary pollution	Microbes have poor tolerance to radiation
Adsorption	Simple operation	High requirements for adsorbent
Photocatalytic	Low cost, high safety, high efficiency, no secondary pollution	Affected by the environment, the charge separation efficiency is low, and the utilization rate of sunlight is low
Combination process	A high degree of purification	High process design requirements and high operating costs

## Data Availability

No new data were created or analyzed in this study. Data sharing is not applicable to this article.
